# Supplementary material for: Effect of Sugar Beet Pulp on the Composition and Predicted Function of Equine Fecal Microbiota
Source: Biology (Basel). 2023 Sep 19;12(9):1254. doi: 10.3390/biology12091254 (PMC10525916; doi:10.3390/biology12091254)
Supplement: Supplementary file 1 [file biology-12-01254-s001.zip › biology-2581104-supplementary.pdf]

Supplemental Figures

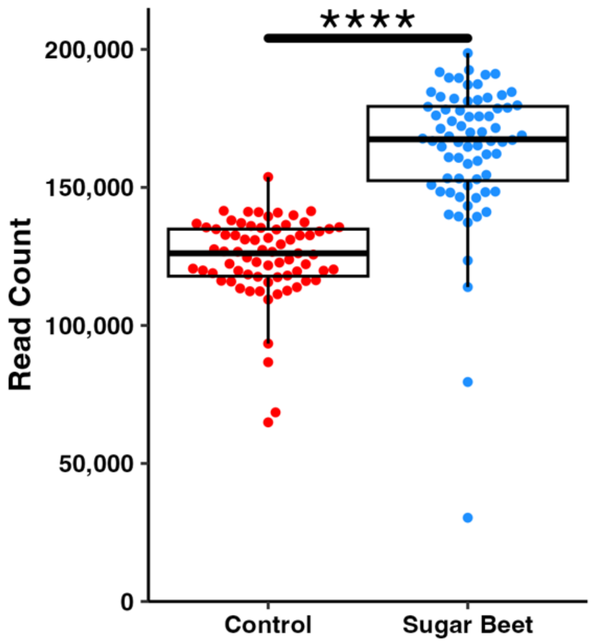

Figure S1. Dot plot showing difference in read counts between control and sugar beet pulp treatment groups. **** $p < 0.0001$, student’s t-test.

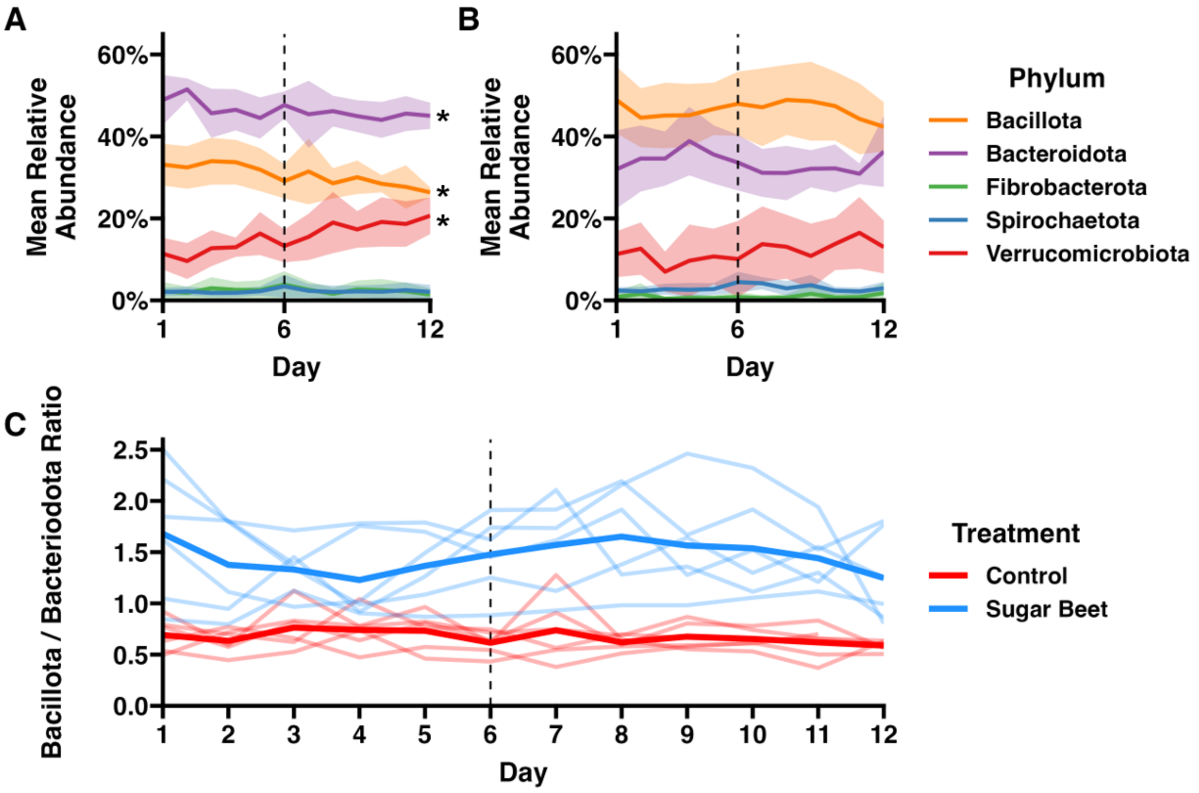

Figure S2. Relative abundance of dominant (relative abundance > 1%) phyla over the course of the study in Control (**A**) and SBP (**B**) groups. Dark line and lighter ribbon denote mean and standard deviation. (**C**) Bacillota-to-Bacteroidota (formerly Firmicutes-to-Bacteroidetes) ratio over time. Vertical dotted line indicates the introduction of SBP to the diet in the treatment group. Time: * $p < 0.05$. One-way ANOVA within phylum.

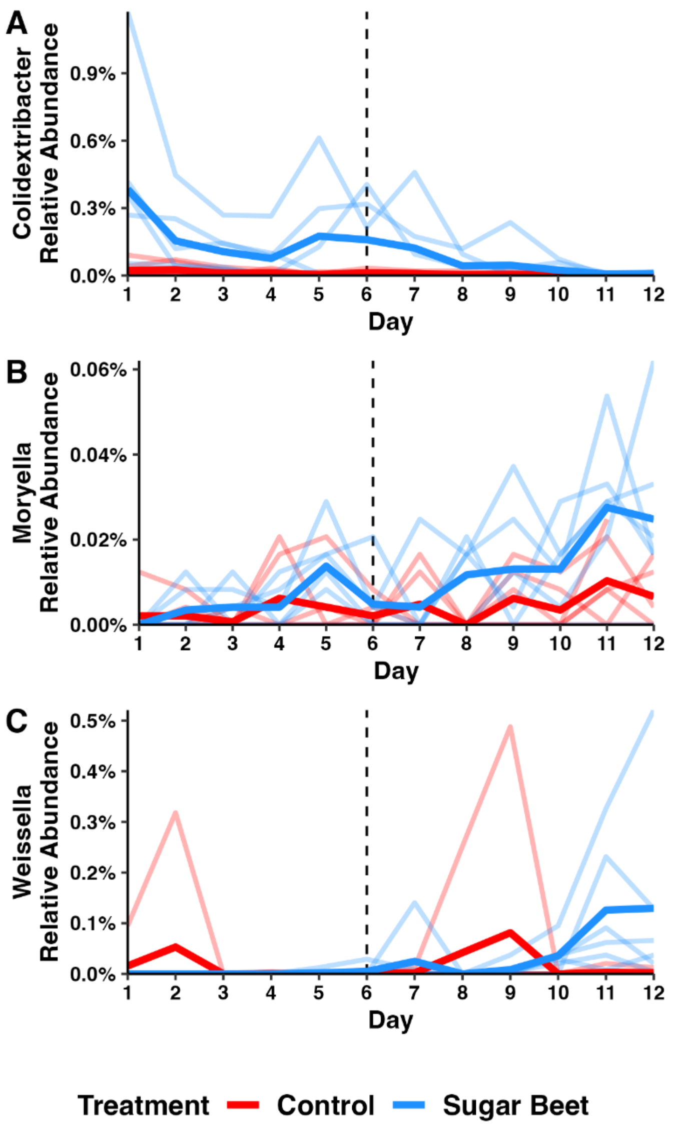

Figure S3. Spaghetti plots depicting relative abundance of **(A)** *Colidextribacter*, **(B)** *Moryella*, and **(C)** *Weissella* across time. Light lines depict individual horses; dark lines depict average relative abundance of groups (legend below). Dotted vertical line indicates diet change for SBP group on day 6 (D6).

Horse	Group	Breed	Sex	Age
Gunner	control	American Quarter Horse	MC	24
Rosie	control	American Paint	F	22
Christy	control	American Quarter Horse	F	11
Ellie	control	American Quarter Horse	F	18
Pride	control	Thoroughbred	MC	14
Diamond	control	American Quarter Horse	F	18
Diamond	sugar beet pulp (SBP)	American Quarter Horse	F	19
Cricket	sugar beet pulp (SBP)	Pony-Unlisted Breed	F	22
Frosty	sugar beet pulp (SBP)	American Quarter Horse	MC	19
Atlas	sugar beet pulp (SBP)	Thoroughbred	MC	11
Albie	sugar beet pulp (SBP)	American Paint	MC	19
Quest	sugar beet pulp (SBP)	American Paint	MC	19

Table S1. Demographics of horses used in each treatment group
F = female, MC = male castrated; age in years
